# Data set for effect of cetane enhancer on ceramic coated diesel engine fuelled with neat Moringa oleifera methyl ester

**DOI:** 10.1016/j.dib.2019.103932

**Published:** 2019-04-19

**Authors:** V. Karthickeyan

**Affiliations:** Department of Mechanical Engineering, Sri Krishna College of Engineering and Technology, Kuniamuthur, Coimbatore, 641 008, Tamil Nadu, India

**Keywords:** *MOME*, Diesel engine, Cetane enhancer and ceramic engine

## Abstract

The present data article is based on the research work which investigates the effect of cetane enhancer on thermally coated engine fuelled with *Moringa oleifera methyl ester (MOME)*. In this experimental work, Kirloskar TV1 model direct injection water cooled diesel engine with eddy current dynamometer was used. *MOME* was produced by two-stage transesterification process. The physio-chemical properties of *Moringa oleifera methyl ester (MOME)* were analysed based on American Standards for Testing Materials (ASTM) standards and data's were presented. Further, the fuel properties were enhanced with the addition of 1% of cetane enhancer (namely Pyrogallol) to MOME and data's related to improved fuel properties were presented.

Engine was loaded from minimum load to maximum load using eddy current dynamometer. The combustion chamber components such as piston head, cylinder head and intake and exhaust valves were coated with Yttria Stabilized Zirconia (YSZ) to transfigure the normal engine into low heat rejection engine. Engine tail pipe emissions were determined using AVL, Austria make 444 di-gas analyser and AVL, Austria make 437C smoke meter equipment. Data related to fuel samples like diesel, *MOME* with and without Cetane enhancer in normal and ceramic engines were presented.

**Table:** Specifications Table

Subject area	*Alternate fuels, IC Engines*
More specific subject area	*Biodiesel, Cetane enhancer, Thermal barrier coating*
Type of data	*Figures, tables and graphs*
How data was acquired	*Engine performance data were measured using computerized Kirloskar TV1 model four stroke direct injection diesel engine. Emission characteristics like carbon monoxide, hydrocarbon and oxides of nitrogen were measured using AVL 444 di-gas analyser and smoke was measured using AVL 437C smoke meter.*
Data format	*Raw and tabulated.*
Experimental factors	*Performance characteristics of Moringa oleifera methyl ester depends on the physio-chemical properties like calorific value, cetane number, flash point, kinematic viscosity and density. Engine emission characteristics of Moringa oleifera methyl ester depends on oxygen presence, CHO (Carbon, Hydrogen and Oxygen) and fuel viscosity.*
Experimental features	*Raw Moringa oleifera oil was converted into Moringa oleifera methyl ester using two-stage transesterification process. The physio-chemical properties of MOME were analysed based on ASTM standards. In addition, the fuel properties were improved with the addition of 1% of cetane enhancer namely Pyrogallol (PY) to biodiesel. Low heat rejection was achieved by coating the combustion chamber components using YSZ ceramic material.*
Data source location	*10°56′09.9″N 76°57′21.8″E*
Data accessibility	*Data is with this article.*
Related research article	*V. Karthickeyan, Effect of cetane enhancer on Moringa oleifera methyl ester (MOME) in a thermal coated direct injection diesel engine, Fuel. 235 (2019) 538–550.*https://doi.org/10.1016/j.fuel.2018.08.030

**Table:** 

**Value of the Data** • The data reported the technical feasibility and eco-friendly prospective of *Moringa oleifera methyl ester (MOME)* as the most prominent alternative fuel to the conventional diesel. • The data provided the immense support to scientific community as the physical and chemical properties of *MOME* were analysed based on ASTM standards and compared with diesel. • The data can be used to investigate the effect of pyrogallol on *MOME* and offer information about the engine characteristics to the scientific community. • The data conveyed the significance of converting the normal diesel engine into ceramic coated engine and gains more attraction over wide research community. • The data can be used by the research community to compare the performance and emission characteristics of *MOME* and Pyrogallol in normal and ceramic coated engine.

## 1 Data

**Fig. 1:**
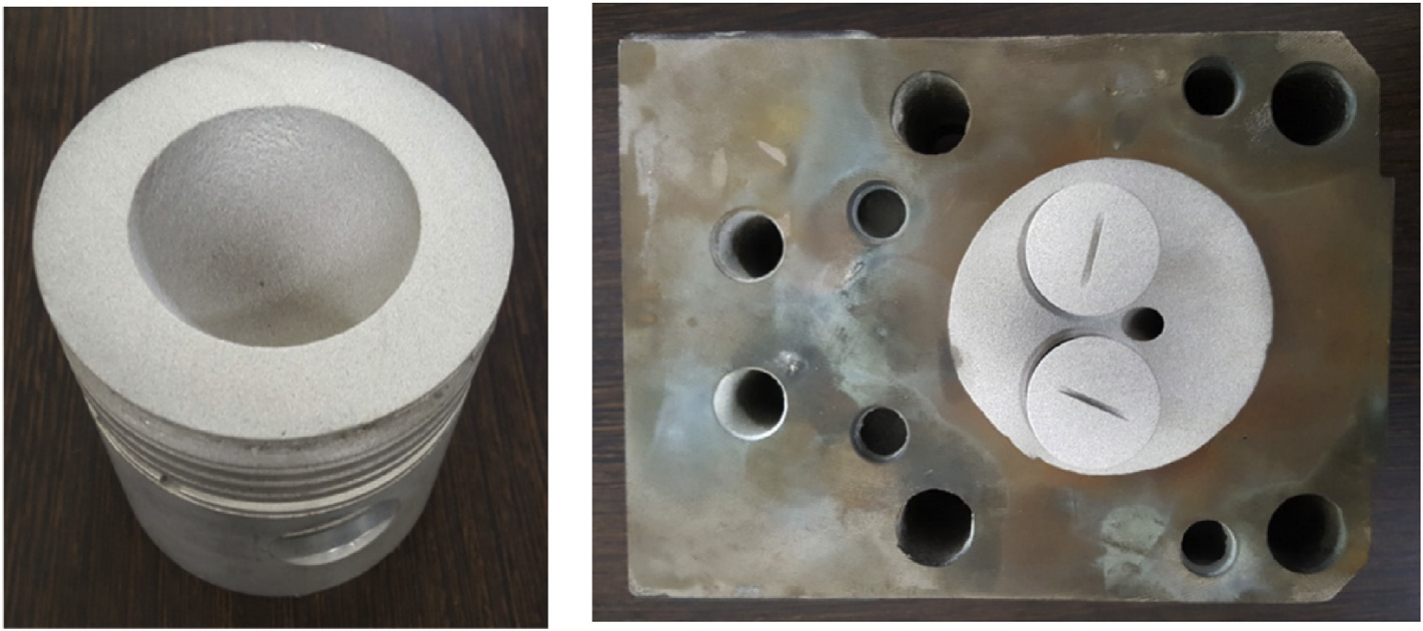
Yttria stabilized zirconia coated engine components.

The data presented in this article was based on the experimental study on the effect of cetane enhancer on *Moringa oleifera* methyl ester in normal and ceramic coated engine. Table 1 represents the data regarding fuel properties of diesel, MOME and MOME+PY based on ASTM testing procedure. Fig. 1 represents the Yttria stabilized zirconia coated engine components. Table 2 provides the data information about brake thermal efficiency of engine in terms of % for diesel and MOME+PY in normal and ceramic coated engine at various loads (20, 40, 60, 80 and 100%). Table 3 provides the data information about brake specific fuel consumption in terms of kg/kWh for diesel and MOME+PY in normal and ceramic coated engine at various loads (20, 40, 60, 80 and 100%).

**Table 1:** Properties of fuel.

Property	ASTM standards	Diesel	MOME	MOME+PY
Density (kg/m^3^)^a^	D1298	835.1	859.3	839.42
Kinematic viscosity at 40 °C (cSt)^a^	D445	2.57	5.05	3.21
Flash point (°C)^a^	D93	56	150.1	91
Fire point (°C)^a^	D93	62	162	95
Gross calorific value (MJ/kg)^a^	D240	43.26	40.06	42.33
Cetane number	D613	48	56	62
C (mass %)		–	76.32	–
H (mass %)		–	12.21	–
O (mass %)		–	11.46	–
C/H		–	6.25	–

a All properties were determined based on ASTM standards under laboratory condition.

**Table 2:** Brake thermal efficiency in-terms of % at all loads.

Load (%)	Normal engine		Ceramic engine	
	Diesel	MOME+PY	Diesel	MOME+PY
20	5.9	5.5	6.3	6.6
40	12.2	11.6	13.1	13.7
60	16.7	16	17.8	18.5
80	20.1	19.8	21.9	22.8
100	26.9	26.3	27.3	28.1

**Table 3:** Brake specific fuel consumption in-terms of kg/kWh at all loads.

Load (%)	Normal engine		Ceramic engine	
	Diesel	MOME+PY	Diesel	MOME+PY
20	1.48	1.53	1.37	1.44
40	0.96	1.05	0.88	0.86
60	0.64	0.71	0.56	0.51
80	0.52	0.55	0.41	0.38
100	0.39	0.43	0.32	0.29

**Fig. 2:**
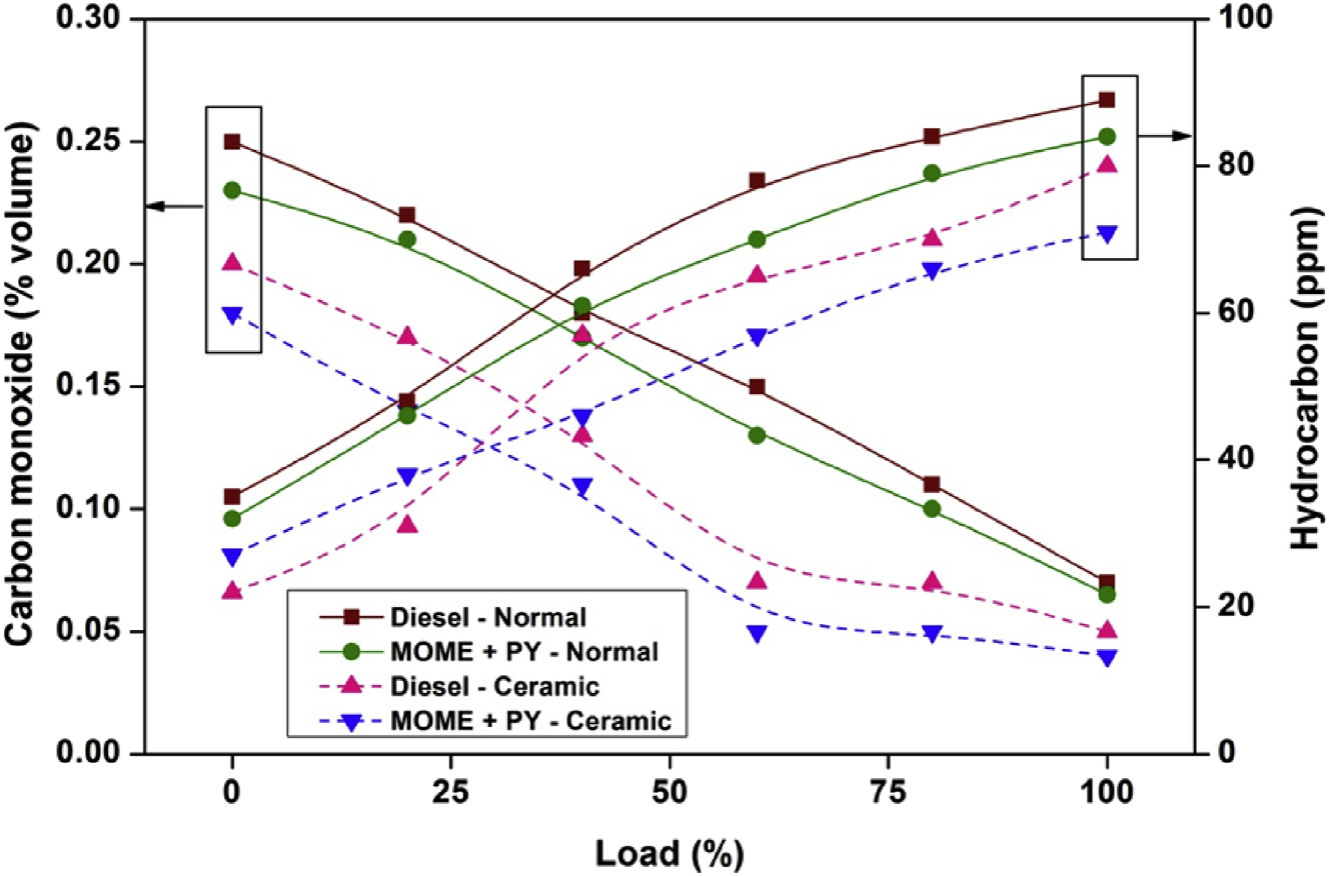
Variation of carbon monoxide and hydrocarbon against various engine loads.

**Fig. 3:**
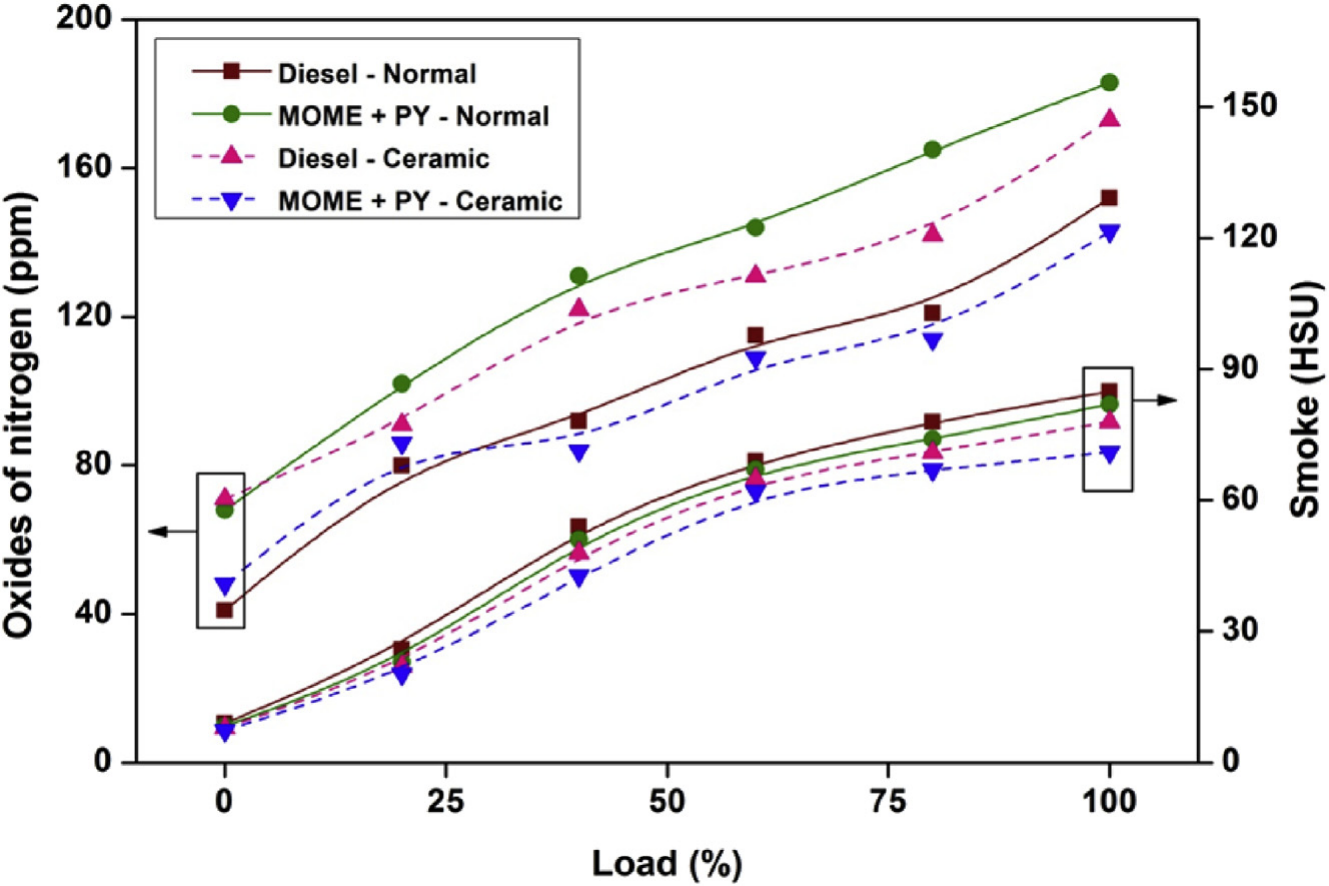
Variation of oxides of nitrogen and smoke against various engine loads.

Fig. 2 depicts the carbon monoxide and hydrocarbon emissions at different engine loads for diesel and MOME+PY in normal and ceramic coated engine. The variation of oxides of nitrogen and smoke emissions for diesel and MOME+PY in normal and coated engine was shown in Fig. 3.

## 2 Experimental design, materials, and methods

Raw *Moringa oleifera* oil was converted into *Moringa oleifera* methyl ester using two-stage transesterification process. As acid value of raw oil was high, two stage esterification process was performed [4], [5], [6]. Acid esterification was carried out using 6:1 methanol to oil ratio with the addition of 0.5 (w/w) of H_2_SO_4_ to preheated oil [2], [3]. The solution was stirred with magnetic stirrer for 1 hour at the speed of 600 rpm incessantly. From the separation funnel, bottom layer as taken and processed with methanol and potassium hydroxide at reaction time of 1 hour and stirring speed of 60 minutes. The last derived component from separation funnel was called as crude *Moringa oleifera* methyl ester. The methyl ester was purified with warm de-ionized water for thrice. The properties of MOME were evaluated based on ASTM [1], [7], [8] condition under laboratory condition and blended with 1% of PY to achieve improved fuel properties.

In the present work, diesel was considered as the baseline fuel and MOME was blended with 1% of PY. Both samples were investigated in normal and ceramic coated engine at various loads. Kirloskar make TV1 model direct injection diesel engine with water cooled eddy current dynamometer was used for the experimental analysis. The main specification of engine were bore x stroke of 87.5 × 110 mm, compression ratio of 17.5:1, injection pressure of 210 bar, injection timing of 21° before top dead centre and speed of 1500 rpm. Air and fuel flow unit was attached to the electronic flow control unit and linked with National Instrument (NI) based data acquisition system (DAQ). Similarly, all other electronic components associated with engine were linked with NI-DAQ. Matlab based EngineSoft Version 4.0 was used for online plotting and data recording purpose. Engine tail pipe emission was measured using AVL 444 di-gas analyser. Carbon monoxide was measured in the range of 0–10% volume, hydrocarbon in the range of 0–10000 ppm and oxides of nitrogen in the range of 0–5000 ppm. An AVL 437C smokemeter was used for the measurement of smoke in terms of HSU in the range of 0–100.
